# Supplementary material for: Are carbon emissions trading and green financial instruments synergistic? -Comprehensive quantitative research based on content analysis
Source: PLoS One. 2024 Mar 7;19(3):e0298601. doi: 10.1371/journal.pone.0298601 (PMC10919601; doi:10.1371/journal.pone.0298601)
Supplement: S1 Data — (ZIP) [file pone.0298601.s001.zip › Data/地级市Z_F工作报告.zip!/╔╜╢1⁄2/╤╠╠¿╩╨/└┤╫╘╣1⁄2╓┌║┼í╛┬φ┐╦╩2╛▌═°í┐_2007.docx]

各位代表：

现在，我代表市人民政府向大会作政府工作报告，请予审议，并请市政协委员和其他列席会议的同志提出意见。

一、2006年政府工作回顾

2006年是“十一五”规划的开局之年，是国家宏观调控力度不断加大的一年，也是我市开拓进取、经济社会发展取得重大成就的一年。一年来，在中共烟台市委的坚强领导下，全市上下高举邓小平理论和“三个代表”重要思想伟大旗帜，认真贯彻党的十六大和十六届三中、四中、五中、六中全会精神，以科学发展观统领全局，以“又好又快、赶超发展、走在前面”为目标，解放思想，抢抓机遇，团结奋进，干事创业，改革开放和现代化建设取得了新的成绩，较好地完成了市十四届人大四次会议确定的各项任务，实现了“十一五”时期的良好开局。初步核算，全市生产总值完成2402亿元，比上年增长17%，其中一、二、三次产业增加值分别增长5.9%、20.4%和13.9%。全市地方财政收入完成112.4亿元，增长29.2%。城市居民人均可支配收入14374元，农民人均纯收入6072元，分别增长15.4%和13.6%。

——调整优化产业结构，经济增长方式加快转变。认真贯彻国家宏观调控政策，全面清理新开工项目，集中整顿土地市场，投资规模得到有效控制。固定资产投资完成1591.2亿元，增长19.8%。海阳核电项目取得突破性进展，鸿富泰电脑及主板二期扩产等项目投入建设，东岳汽车二期扩建、南山高精度轻合金等项目相继投产，北方奔驰重型汽车整车下线。大力推动节能降耗，扶持发展循环经济，重点行业、重点领域、重点企业和重点项目的节能降耗成效明显。粮油喜获丰收，农林牧渔业协调发展，农业产业化、标准化、国际化进程不断加快，高致病性禽流感等重大动物疫病防控工作成效明显。全市年销售收入过亿元的农业产业化龙头企业达到71家，70%的农户进入产业化经营领域。工业结构调整力度加大，“3·50工程”全面启动，骨干企业、企业技术中心和名牌产品培育迈出新步伐，机械制造、电子信息、食品加工和黄金四大支柱产业进一步壮大，汽车、手机、电脑三大产品集群渐成规模。去年，全市规模以上工业销售收入、利税、利润分别增长33%、28.2%和31.8%。高新技术产业产值达到1612亿元，增长42.8%。销售收入过10亿元的企业和利税过亿元的企业均达到77户，分别增加29户和16户。新增中国名牌产品7个、中国驰名商标4件。全年实现旅游业总收入147.9亿元，增长25.8%。全市沿海港口货物吞吐量突破1亿吨，其中集装箱吞吐量达到116.9万标箱，跨入全国“十强”行列。烟大铁路轮渡投入试运营，我市交通枢纽城市地位进一步确立。“万村千乡”市场工程顺利推进，一批国内外知名商贸流通企业相继落户我市，城乡市场日趋活跃。全市社会消费品零售总额完成698亿元，增长16.4%。民营经济实现新发展，全市个体工商户达到13.6万户，增长10.7%；私营企业注册资本594亿元，增长27.3%。

——进一步扩大改革开放，发展活力明显增强。企业改革取得新进展，完成41户市属国有企业产权制度改革任务，分流安置了4000多名市属特困企业职工。实施股权分置改革的9户境内上市企业全部完成改革任务，金丰制革、东方海洋、招金矿业3户企业在境内外成功上市，募集资金33.5亿元。农村综合改革扎实推进，乡镇机构改革试点和村级债务化解试点工作顺利完成，农村义务教育经费保障机制改革正式启动，县乡财政体制改革全面展开，81%的村完成了土地延包任务。加大招商引资力度，全面开拓国际市场，加快转变外贸增长方式，继续实施“走出去”战略，对外开放迈上新台阶。成功承办了第八届国际果蔬·食品博览会、亚欧会议旅游合作发展论坛暨展览会、中国国际美食节。台湾农民创业园获得国家批准，出口加工区成为全国拓展保税物流功能试点单位，增设了2个省级开发区和6个国别工业园区，与法国昂热市建立了友好城市关系。全年新批外资项目1086个，实际外商直接投资21.5亿美元，增长13%。实现外贸进出口150.8亿美元，增长31.3%，其中出口88亿美元，进口62.8亿美元，分别增长35.7%和25.6%。对外承包工程营业额和外派劳务分别增长12.4%和12.6%。

——加快社会主义新农村建设，农村生产生活条件持续改善。按照“生产发展、生活宽裕、乡风文明、村容整洁、管理民主”的要求，加快村镇水、电、路、垃圾和污水处理等设施建设，实施农村环境综合整治，加强农村学校、医院、老年公寓等公益事业和社会保障体系建设，积极开展机关事业单位、企业、小康村与一个村“3+1”联手共建新农村活动，实现了新农村建设的良好开局。全面开展村镇规划编制工作，完成了全市村庄布点规划和60%的村镇规划编制任务。全年新增自来水供水村1512个，改造硬化农村公路1692公里，有87%的村通了自来水，96%的村通了油路。农村电网建设改造工程全部竣工。重点造林绿化工程快速推进，全市共完成造林面积0.6万公顷。抗旱双保取得明显成效，胶东调水工程全面开工。有1163个村新建了文化大院，新改扩建敬老院66处，五保对象集中供养率达到80%。培训农民2万多人，带动农村劳动力转移10万多人。

——强化城市规划建设管理，“五年大变样”目标全面实现。健全了城市规划三级评审制度和社会公示制度，完成了新一轮城市总体规划修编、“十一五”城市近期建设规划、烟台海岸带规划和一批专项规划。集中力量推进城市建设重点工程，国际博览中心、体育公园二期工程、成龙线改建、幸福南路拓宽改造等工程全面完工，烟台港西港区一期工程、机场飞行区改扩建、港站区片改造、龙烟铁路、红旗路和套子湾污水处理厂扩建、辛安河污水处理厂二期等新建续建工程顺利推进，2个区片旧居民楼“平改坡”、9个社区综合整治、38个社区公园新建改造任务全面完成，新增市区集中供热面积276万平方米，城市公交中巴全部退市。强化环保工作，实施了一批环境保护工程，全市二氧化硫排放减少2.7%，化学需氧量排放降低3.6%。进一步理顺城市管理体制，启动全国数字化城市管理试点工作，提高了城市管理水平。去年，我市荣膺中国投资环境“金牌城市”、全国创新环境十强城市、全国生活质量十强城市、中国旅游竞争力百强城市，城市美誉度不断提高，对外影响力显著提升，广大市民的自信心、自豪感进一步增强。

——全面繁荣各项社会事业，经济与社会协调发展。“创新型城市”建设全面展开，企业技术创新体系建设得到加强，争取国家科技支撑计划项目8项，承担省重大科技专项9项，列入国家重点新产品计划16项，获国家科学技术奖4项、省科学技术奖45项。市院合作取得新进展，成功争取中科院在我市设立烟台海岸带可持续发展研究所。冰轮、登海种业被认定为国家级企业技术中心，张裕和招远金宝被列入国家创新项目计划。全市专利申请量达到3310件，增长48.6%。人才工作取得新成绩，新引进各类人才1.7万多人，其中硕士以上725人。学前教育稳步发展，义务教育不断巩固，高中段教育快速发展，教育质量进一步提高。通过校企合作组建了一批职业教育集团。中国农业大学烟台校区落成招生。建立完善了困难学生救助体系，全市共救助困难学生24.4万人次。卫生事业加快发展，市级卫生监督和疾病预防控制管理体制进一步完善，城乡医疗救助制度全面实施，完成了22个疾病预防控制体系和医疗救治体系建设项目。新型农村合作医疗覆盖面扩大到8个县市区，农民参合率达到85%。文化事业和文化产业进一步繁荣，群众文化蓬勃开展，专业艺术创作和文化遗产保护工作取得新成绩。成功承办了第二十一届省运会和第七届省残运会，均获得金牌第一、总分第一的优异成绩。人口和计划生育工作稳定发展，连续多年保持低生育水平。全面落实就业再就业优惠政策，全年新增城镇就业再就业13.5万人，城镇登记失业率降到3.1%。提高企业离退休人员基本养老金、农村低保和义务兵优待金标准，扩大基本医疗保险和工伤保险药品报销范围，按时足额为各类保障对象落实保障资金43.1亿元。深入开展爱心捐助活动，进一步加大了对困难群体的救助力度。市政府确定的为民服务实事进展顺利。帮扶栖霞和对口支援定陶、聂拉木工作取得阶段性成果。老龄、妇女、儿童和残疾人事业得到加强，广播电视、新闻出版、气象地震、史志档案、双拥共建、人民防空、民族宗教等工作取得新的成绩。

——推进民主法制建设，和谐稳定局面进一步巩固。积极推进政务公开、厂务公开和村务公开，严格落实重大决策、重要事项公开咨询、公开听证和公示等制度。各级政府主动接受人大、政协及各民主党派、工商联和社会各界的监督，高度重视人大代表建议和政协提案的办理工作。启动实施“五五”普法依法治理规划，积极开展法制宣传教育，大力推进基层依法治理，民主法治建设进一步加强。全面开展全国最安全城市“规范建设年”活动，加强社会治安综合治理，完善治安防控体系，深入开展严打整治斗争，社会治安形势持续稳定。加强信访工作，健全矛盾纠纷疏导控制机制，有力促进了社会和谐。强化安全生产监督管理，安全生产秩序进一步好转。全面开展社会主义荣辱观教育，大力弘扬“创新、拼搏、协作、包容”的烟台精神，扎实推进群众性精神文明创建活动，巩固发展了“全国文明城市”成果。

——加强政府自身建设，行政效能不断提高。全市政府系统坚持以“三个代表”重要思想为指导，深入开展“改进机关作风、优化政务环境”活动，营造了开拓创新、干事创业的浓厚氛围，增强了政府的公信力和执行力。以贯彻实施《公务员法》为契机，修订《政府工作规则》，完善政务运行制度，全面推行行政执法责任制，进一步提高了依法行政水平。严格遵守领导干部廉洁自律各项规定，认真查处违纪违法案件，深入开展行风评议和纠风专项治理，坚决纠正损害群众利益的不正之风，进一步树立了政府机关和公务员队伍的良好形象。

各位代表，过去一年所取得的成绩，是上级党委、政府和中共烟台市委正确领导的结果，是人大、政协和社会各界监督支持的结果，是全市人民同心同德、顽强拼搏的结果。在此，我代表市人民政府，向全市广大工人、农民、知识分子、各级干部以及各阶层的建设者，向驻烟人民解放军、武警部队官兵和政法干警，向中央、省属驻烟单位，向各位人大代表和政协委员，向各民主党派、工商联、无党派人士和各社会团体，向所有关心支持烟台建设的港澳同胞、台湾同胞、海外侨胞和国际友人，致以崇高的敬意和衷心的感谢！

各位代表，我市经济建设和社会发展虽然取得了很大成绩，但也存在不少问题和困难，主要表现在：经济发展的支撑体系不够牢固，结构性矛盾比较突出，农业基础地位比较薄弱，服务业发展相对滞后，民营经济发展潜力挖掘不够；自主创新能力不够强，节能降耗形势依然严峻，生态环境比较脆弱，环境保护压力较大；县域经济发展不够平衡，部分县乡财政存在困难，农民增收难度较大；体制机制性障碍依然存在，市场化程度有待进一步提高；社会事业发展相对较慢，社会保障制度需要进一步完善，一些群众关心的问题解决得还不够好，影响社会和谐稳定的因素仍然不少，构建和谐社会任重道远；政府自身建设需要进一步加强，少数地方和部门工作作风不实，工作效率不高，个别机关工作人员为政不廉，腐败现象依然存在。对于这些问题，我们一定高度重视，下决心认真加以解决。

二、2007年政府工作总体安排

2007年是落实科学发展观、构建和谐社会的重要一年，也是巩固宏观调控成果、保持良好发展势头的关键一年。综合分析国内外形势，我们既面临难得机遇，也面临严峻挑战。面对新形势、新任务、新挑战，我们必须进一步增强机遇意识、危机意识和创新意识，解放思想、与时俱进，奋发有为、乘势而上，努力开创经济社会发展新局面。今年的政府工作，要坚持以邓小平理论、“三个代表”重要思想为指导，深入贯彻党的十六大和十六届三中、四中、五中、六中全会精神，全面落实科学发展观，认真执行国家宏观调控政策，积极调整经济结构，加快转变增长方式，纵深推进改革开放，着力提高自主创新能力和节能环保水平，注重解决好“三农”问题，切实保障人民群众的切身利益，加快构建社会主义和谐社会，努力推动经济社会又好又快发展。

今年全市经济社会发展主要预期目标为：生产总值增长14%；地方财政收入增长20%；固定资产投资增长18%；社会消费品零售总额增长15%；实际外商直接投资增长12%，外贸进出口总额和外贸出口均增长17%；城市居民人均可支配收入增长10%，农民人均纯收入增长8%；城镇登记失业率控制在3.3%以内，人口自然增长率控制在2.5％。以内；万元生产总值能耗下降4.5%，二氧化硫排放减少7%，化学需氧量排放降低5%。在工作指导上，坚持“四个着力”：

——着力调整经济结构和转变增长方式。加快调整三次产业结构，膨胀支柱产业，扶持骨干企业，培植名牌产品，改造提升传统产业，发展壮大高新技术产业。加快发展城市经济，扶持发展农村经济，做到工业反哺农业、城市支持农村，城乡互动，共同发展。推进经济增长由粗放型向集约型转变，实现速度、质量、效益相协调，消费、投资、出口相协调，人口、资源、环境相协调。

——着力加强资源节约和环境保护。高度重视节约能源资源、集约用地和保护生态环境，控制、改造和淘汰落后生产能力，完善市场调节机制，加强依法治理，推进节能降耗，减少污染物排放。大力开展造林绿化，改善生态环境，推进生态市建设。注重发展循环经济，鼓励企业开发利用先进实用的节能新技术、新产品，加快建设资源节约型、环境友好型社会。

——着力推进改革开放和自主创新。深化国有企业、投资、财税、金融、物价、事业单位和行政管理体制改革，力争在重点领域和关键环节取得突破，加快形成贯彻落实科学发展观的体制机制保障。积极适应入世过渡期结束后的新形势，坚定不移地实施经济国际化战略，坚持以开放促改革、促调整、促发展，努力拓展开放领域，提升开放层次，提高开放质量，开创对外开放新局面。更加重视科技进步与自主创新，鼓励企业和全社会走自主创新道路，提高开放条件下的原始创新能力、集成创新能力和引进消化吸收再创新能力，加快建设创新型城市。

——着力促进社会发展和解决民生问题。坚持经济社会协调发展，优先发展教育事业，加强医疗卫生服务，加快发展文化事业和文化产业。坚持以人为本，把不断改善人民生活作为经济社会发展的根本目的，千方百计提高城乡居民生活质量和水平。继续抓好市政府为民服务实事工程，切实解决人民群众最关心、最直接、最现实的利益问题，维护社会公平正义，让广大人民群众共享发展成果。

三、努力推动经济又好又快发展

（一）认真贯彻国家宏观调控政策，推动经济增长转入科学发展轨道。把贯彻落实国家宏观调控政策作为推动经济又好又快发展的根本保证，坚持经济增长与资源节约、环境保护相统一，坚持有保有压相结合，严把土地、资金闸门和市场准入门槛，强化投资项目管理，坚决控制高耗能、高污染和产能过剩、盲目扩张项目，鼓励扶持符合国家产业政策和我市实际的重点项目，引导投资向高新技术产业、先进制造业、现代农业、现代服务业和社会事业倾斜。全面开展“项目推进年”活动，全力推进90个重大项目建设，形成一批新的支撑点和增长点。

（二）膨胀提升先进制造业，促进工业结构优化升级。进一步强化工业立市、工业强市观念，不断优化工业行业结构、企业结构和产品结构，大力膨胀机械制造、电子信息、食品加工和黄金四大支柱产业，积极培植石化、冶金、生物制药三个潜力产业，做大做强汽车、电脑、手机三大产品集群，加快发展船舶、特种化纤、葡萄酒等产品集群。深入实施“3·50”工程，加大骨干企业培育力度，加强企业技术改造与自主创新，形成一批具有自主知识产权的主导产品和关键技术，培育一批在国内外市场具有较高知名度和影响力的名牌产品，提高我市制造业的核心竞争力。年内，力争销售收入过20亿元的企业达到35户，其中过百亿元的3户以上；力争再创建省级以上技术中心3家；力争新增中国名牌产品4个、中国驰名商标2件；力争工业投入增长18%，技改投入增长20%。推进市属国有企业投资主体多元化改造、主辅分离和辅业改制，优化国有经济布局，增强企业发展活力，确保国有资产保值增值。加大企业上市工作力度，提高从资本市场直接融资的能力，加快中小企业境外上市步伐，推进烟台氨纶、富海实业等企业尽快上市，做好清泉实业、恒邦冶炼等一批后备企业上市工作，促进上市公司融资扩张。以实施“353示范工程”为切入点，大力发展循环经济。以10大节能工程和30个投资过千万元的节能改造项目为切入点，继续抓好节能降耗，加强高耗能行业和110户重点用能企业的节能管理。高度重视环境保护和清洁生产，抓好电力行业脱硫、污水处理厂建设和水泥行业结构调整。

（三）突破发展现代服务业，增强消费对经济的拉动力。坚持生产性服务业与生活性服务业并重，大力发展旅游会展、现代物流、商贸餐饮和金融保险业，积极培育信息服务、文化传媒和社区服务业，加快重点项目建设，膨胀壮大骨干企业，推动服务业上层次、上水平。整合全市旅游资源，构筑蓬莱、长岛、龙口和市区“3+1”主体板块，拓展旅游产业链，启动创建中国最佳度假旅游城市工作。发挥港口、铁路、公路、机场等优势，以口岸物流、保税物流、制造业物流、城市配送物流为重点，大力发展现代物流业，推动我市与东三省及环渤海地区的交流与合作。围绕繁荣商贸餐饮业，优化城市商业功能布局，培育骨干流通企业和大型批发市场，打造特色商贸集聚区，发展新型流通业态，做大特色餐饮品牌，大力发展城市夜间经济，深入抓好“万村千乡”市场工程。积极推进金融改革开放，优化金融环境，建立金融协调机制，引进中外金融机构，发展壮大地方金融业，充分发挥金融对经济发展的服务作用。年内，第三产业增加值完成843亿元，增长16%。

（四）积极发展现代农业，扎实推进社会主义新农村建设。把发展现代农业、增加农民收入作为推进新农村建设的首要任务，加大投入力度，构筑产业体系，提升装备水平，强化科技支撑，不断提高农业综合效益和竞争力。在保证全市基本农田、基本粮田和粮食总产稳定的前提下，调整优化种植业结构，大力发展优质水果和无公害绿色有机蔬菜，积极培育种子、花卉和食用菌产业，做大做强水产和畜牧业，继续抓好重大动物疫病防控。加强农业综合开发，加大农村基础设施建设投入，建设一批农业设施大工程、产业化大项目，增强对农业农村发展的支撑带动力。纵深推进农业产业化经营，培植大宗优势农产品生产基地，鼓励发展各类新型专业合作组织和农业产业化龙头企业。从今年开始到2010年，市财政每年安排1000万元用于支持发展烟台苹果、烟台大樱桃和莱阳梨，安排500万元用于培育百强龙头企业。推行农业标准化生产，实行市场准入制度，不断提高农产品质量安全水平。组织实施农业重大技术攻关，加快科技成果转化，积极引进新品种、新技术、新机械和先进管理经验，改造传统农业，加快农业水利化、机械化和信息化进程。大力发展农村二三产业，拓展农村发展、农民增收的新空间。以建设“生态城市、绿色烟台”为目标，重视保护和发展森林资源，全年成片造林10万亩。坚持工业反哺农业、城市支持农村的方针，全面落实各项支农惠农政策，加大农村基础设施建设投入，建立健全财政支农资金增长机制，确保财政支农投入的增量继续高于上年，固定资产投资用于农村的增量继续高于上年，土地出让收益用于农村建设的增量继续高于上年。扩大良种补贴、农机具购置补贴和农村沼气建设补贴的数量和范围。加快推进村村通自来水工程，进一步抓好农村主街道硬化美化，开展创建农村文明一条街活动，推进村级组织活动场所建设，集中开展沼气综合开发利用工程。抓好500个村庄整治试点，带动更多的村庄实施综合整治。深化乡镇机构改革、农村义务教育综合改革、县乡财政管理体制改革、农村征地制度改革、农村社区产权制度改革和农村金融体制改革，积极探索化解乡村债务的有效途径，全面推行农村财务委托代理制度，逐步实行村级干部工资县乡统筹。坚持农村土地基本经营制度不动摇，积极稳妥地搞好土地延包，促进土地合理流转和土地资源优化配置，做好被占用土地农民的生活和就业保障工作。启动“村企联手共建年”活动，推进“3+1”联手共建新农村活动深入开展。

（五）优先发展高新技术产业，不断提高核心竞争能力。抓好以企业为主体的技术创新体系和以科研院所、大专院校为主体的知识创新体系建设，引导企业增加研发投入，支持驻烟高校开设适应烟台产业发展的优势学科，鼓励企业与高校院所进行科技合作开发，创建重点实验室和产学研联合体，加快中科院烟台海岸带可持续发展研究所建设。抓好以中介机构为主体的创新服务体系建设，积极推进烟台大学三校科技园、留学人员创业园和烟台软件园建设。抓好科技资源共享平台和科技信息服务平台建设，继续实施科技自主创新工程，培养科技自主创新龙头。实施一批高新技术产业重大科技项目，加快高新技术产业化和传统产业高新化步伐，用高新技术引领先进制造、新材料、电子信息、生物与制药等优势产业发展。全年高新技术产业产值达到1950亿元，增长21%。

（六）全面发展外向型经济，在更广领域、更高层次上参与国际竞争与合作。着力提高利用外资质量。以园区和骨干企业为载体，大力开展产业招商和专业招商，加强与世界500强和国外大公司、大商社的合资合作，扩大外资并购、境外上市融资和现有外资企业增资扩股。推进外贸增长方式转变。继续实施科技兴贸和出口品牌战略，支持机电产品和高新技术产品出口，引导加工贸易转型升级，开拓多元化国际市场，扩大紧缺资源、先进技术和关键设备进口。引导企业运用国际规则发展对外贸易，化解贸易摩擦，提高应对国际市场风险能力。更加积极有效地实施“走出去”战略。支持有条件的企业到境外投资，开发紧缺资源，建立生产销售基地，开展对外工程承包与劳务合作。优化提升对外开放环境。实施“大通关”战略，推进海关、商检和其他口岸联检服务，整合完善口岸电子网络功能，提高通关效率。增加机场、港口国际航线航班，提高烟台口岸承载力。加快建设国际产业转移配套社区，解决外商就医和子女入学等方面的实际问题。用好出口加工区功能升级政策，拓展保税物流业务，实施港区联动，推动整体发展。加强与东亚环黄海城市合作交流，办好“第九届国际果蔬·食品博览会”和“中国烟台国际葡萄酒节”。

（七）放手发展民营经济，大力培植新的经济增长点。把民营经济作为事关全市经济长远发展的重要潜力点和增长点，认真落实国家和省、市各项扶持政策，放宽市场准入，改善融资条件，加快构建融资担保、信用评价、人才培训、技术信息和法律维权服务体系，实施银企联合、中小企业成长、特色产业提升计划，开展创建民营经济强县（市、区）、强乡（镇）、强企活动，引导民营企业加强管理，培育一批原创型企业，壮大一批骨干企业，形成一批特色产业集群，争取全市民营经济增加值增长18%。

（八）加快发展海洋经济，拓展新的发展空间。深入研究论证海洋经济发展规划，确定功能区划，搞好产业布局，抓好渔业资源保护和开发。启动建设一批海洋经济龙头项目，加快发展海上养殖、远洋捕捞、海洋运输、海洋旅游、海洋化工、海洋生物、海洋环保等特色鲜明的海洋产业。突出发展港口经济，加强港口基础设施建设，重点推进烟台港西港区、龙口港10万吨级散货泊位、莱州港扩建和输油管道等工程建设，抓好各港口运输通道设施改造，改善港口集疏运条件。进一步优化港口布局，合理配置港口资源，推动各港口企业有序竞争和协调发展。扶持临港工业，建设临港物流园区，培植骨干企业，全力推进大宇造船等重点项目建设，推动港口经济快速发展。

（九）整体打造城市经济，增强中心城市承载力和辐射力。创新城市发展思路。强化大城市、大市区理念，坚持同城统筹、共兴共荣，把五区作为一个整体，统筹规划发展空间，整合优化资源配置，合理布局产业重点，推进各区在管理体制、交通体系、基础设施、配套政策等方面的对接与融合，实现一体化发展。加快发展城市经济。完善发展芝罘区、莱山区，提升服务业层次和城市管理水平；加快发展福山区、牟平区，着力推进工业化和城市化进程；突出开发区龙头带动作用，打造全市产业高地和先进制造业中心；培植壮大市属重点企业，强化骨干企业带动能力。依托中心市区在区位、港口、科教、人口等方面的优势，积极发展港口经济、高新技术产业和现代服务业，推动城市经济向高端化发展，提高城市经济在全市经济中的比重，增强中心城市对县域和周边的辐射带动能力。着力提升城市形象。围绕建设半岛城市群和制造业基地中心城市、环渤海经济圈骨干城市、区域性交通枢纽城市和滨海度假旅游城市，进一步加强城市规划建设管理。抓好新一轮城市总体规划和“十一五”城市近期建设规划的实施，尽快形成市区滨海带状城市结构。加快推进市区红旗东西路、学院路、通世南路延伸、青年南路延伸、芝罘岛滨海路等在建工程，启动建设以南大街为轴线的沿街开发项目，规划建设烟台商业步行街，加快建设新青少年宫，进一步配套完善体育公园。继续抓好旧城区环境综合整治，启动幸福区片整体开发改造工程，加强公园、公厕、停车场、过街通道等便民设施建设和管理，保护和利用好近代建筑。优先发展城市公交，完善公交场站和公交专用道，改善市区交通拥挤状况。加强城市重大基础设施建设，突出抓好火车站改造、烟台港西港区、德龙烟（德州-龙口-烟台）铁路、机场飞行区改扩建、市区大南山一期工程、辛安河污水处理厂二期工程、套子湾污水处理厂二级处理扩建、胶东地区引黄调水、海阳核电等重点项目建设。进一步完善城市管理体制，强化城市综合执法，抓好国家数字化城市管理试点，推广网格化管理，提高城市管理效能。搞好城市品牌经营。用足用好“国际葡萄·葡萄酒城”、“全国文明城市”、“最佳中国魅力城市”、“中国投资环境金牌城市”、“全国社会治安综合治理优秀城市”、“联合国人居奖”等品牌资源，努力把品牌效应转化成经济社会效应。

（十）做大做强县域经济，促进区域经济协调发展。强化政策扶持，完善考核体系，赋予县市更大的自主权，激励各县市因地制宜，发挥优势，走经济特色化、产业规模化、产品集群化的路子，培植主导产业和骨干企业，壮大财政实力。膨胀县级城市，搞好中心镇建设，建设一批综合实力强、聚集程度高、辐射作用大的小城市和中心镇，使之成为带动县域和镇域经济发展的有效载体。鼓励龙口、莱州、招远、蓬莱放大优势，领先发展，不断提升在全国百强县和全省30强县中的位次；促进莱阳、海阳高点定位，赶超发展，及早挤进全国百强县行列；支持长岛立足优势，突出特色，加快建设山东海上明珠和中国最美海岛；帮扶栖霞挖掘资源潜力，用好扶持政策，发展民营经济，做大生态旅游，加强财源建设，实现快速发展。

四、加快构建和谐社会

（一）加强就业和社会保障，维护人民群众切身利益。认真落实促进就业再就业的财税金融政策，鼓励发展就业容量大的产业，支持自谋职业、自主创业，千方百计增加就业岗位。抓好城乡统筹就业试点，建设市级人力资源市场，继续实施农民培训工程，强化对毕业生的就业指导和服务，加强困难群体再就业工作，重点解决好零就业家庭的就业问题。年内，城镇新增就业再就业12万人以上，农村劳动力转移就业10万人以上。建立完善企业工资分配支付制度和工资集体协商制度，依法处理各种劳动争议，有效维护劳动者合法权益。以非公有制经济从业人员、灵活就业人员和农民工为重点，扩大社会保险覆盖范围，加大社会保险费征缴和基金监管力度，确保按时足额发放和基金安全。健全农村养老保险制度，对投保农民由村集体和政府给予适当补助。妥善处理国有改制企业历史遗留问题，保障职工正当权益。认真解决在农村土地征收征用、城市房屋拆迁中侵害群众利益的问题和拖欠农民工工资问题。完善城乡低保制度，逐步提高城乡低保标准。健全农村五保供养体制，五保对象集中供养率保持在80%以上。加强社会救助工作，深入开展爱心捐助活动，大力发展社会福利和慈善事业。完善经济适用住房建设机制，全面建立城市最低收入居民家庭廉租住房保障制度，解决好城市中低收入家庭和困难家庭的住房问题。

（二）繁荣发展各项社会事业，推动社会全面进步。加大科技投入，确保财政科技投入增幅高于财政经常性收入增幅。积极争取更多项目列入国家科技支撑计划、“863”计划、国家重点新产品计划和省重大攻关项目，夺取一批国家和省科技成果奖。重视和搞好科技成果转化，搞好高新区建设。加强知识产权保护，加快专利开发和应用。实施人才强市战略，培养和引进一批高层次人才。认真落实《全民科学素质行动计划纲要》，努力提高全民科学素质。继续把教育事业放在优先发展的位置来抓，逐步将义务教育全面纳入公共财政保障范围，继续发展学前教育，巩固提高义务教育，加快普及高中段教育，积极发展高等教育，大力发展职业技术教育，鼓励发展民办教育，不断提高教育质量和水平。从今年开始，免除城乡义务教育阶段学杂费，对城乡贫困家庭学生免费提供教科书，并补助寄宿生生活费。深化医疗卫生体制改革，健全城乡卫生服务体系，完善各类急诊急救应急预案和公共卫生监测预警机制，加强社区卫生服务，加快实施城乡医疗救助制度，进一步规范医疗收费标准，降低医疗费用。全面推行新型农村合作医疗，各级财政补助标准达到人均40元，实现“新农合”全部覆盖农村的目标。落实传染病、地方病综合防治措施，确保重点疫病不发生暴发流行。加强食品、药品监管，保证人民群众饮食和用药安全。完善市县乡村四级文化服务网络，加快建设烟台大剧院、市京剧院新院、市群众艺术馆新馆，积极筹建市博物馆新馆，加强文化遗产保护，推进文化大市、文化强市建设。深入实施广电“三通”工程，加快广播电视数字化进程。深入开展全民健身活动，加强居民小区体育健身设施建设，积极备战第六届全国城市运动会。进一步做好人口计划生育工作，稳定低生育水平，提高出生人口素质，优化人口结构。积极发展妇女儿童、残疾人和老龄事业，大力发展新闻出版事业，抓好外事侨务、气象地震、民族宗教、史志档案和仲裁工作，加强国防教育、国防动员、民兵预备役和双拥共建工作。

（三）提高社会管理水平，努力维护社会稳定。加强社区建设和管理，完善公共服务设施，推进社区工作者职业化、专业化，强化社区物业管理和综合服务，建设新型和谐社区。积极培育各类农村经济协会、行业协会和社区民间组织。建立健全社会应急管理体制，推进防空防灾一体化建设，完善应急救援预案，有效应对各类突发事件。整顿规范市场秩序，严厉打击走私贩私和假冒伪劣、价格欺诈、强制消费等不法行为。建立企业、个人和中介机构诚信系统，加大失信处罚力度，优化信用环境，建设“诚信烟台”。深入开展创建全国最安全城市活动，进一步加强社会治安综合治理，坚决打击各类犯罪行为，维护国家安全和社会稳定。抓好人民群众来信来访工作，及时预防化解各类社会矛盾。严格落实安全生产责任制，切实加强安全生产监管、执法检查和综合整治，防止重特大事故发生。

（四）加强民主法制建设，推动精神文明建设再上新水平。完善村民自治和城市居民自治机制，加快发展社会主义基层民主政治，依法保障人民群众的民主权利。认真实施“五五”普法依法治理规划，强化法律服务和法律援助，营造良好的法治环境。加快建设社会主义核心价值体系，积极构建和谐文化。深入贯彻《公民道德建设实施纲要》，切实加强青少年思想道德建设，广泛开展社会主义荣辱观实践活动和群众性精神文明创建活动，进一步巩固“全国文明城市”成果。

五、加强政府自身建设

全面落实科学发展观、加快构建社会主义和谐社会，对政府队伍提出了新的更高要求。我们必须进一步强化执政为民理念、依法行政理念和优质高效理念，不断提高驾驭市场经济能力、推进自主创新能力、管理社会能力和依法办事能力，努力建设法治政府、服务政府、阳光政府和高效廉洁政府。

坚持开拓创新，努力提升思想境界。深入学习马列主义、毛泽东思想、邓小平理论和“三个代表”重要思想，全面理解、准确把握中央关于经济社会发展的指导方针和一系列战略决策，不断增强贯彻落实科学发展观的自觉性和坚定性，用科学发展观武装头脑、指导实践、检验工作，正确处理“好”与“快”、“保”与“压”、投资与消费、发展与改革、局部与全局、当前与长远等方面的关系，切实把各方面的积极性引导到科学发展上来。推进学习制度化，努力建设学习型政府。从今年开始，市政府定期进行集体学习，请有关专家学者就科学理论、法律法规、国内外政治经济形势、现代科学知识等方面的问题开设专题讲座，进一步优化知识结构、提高行政水平，做到在认识上有新提高，政策上有新举措，实践上有新进展。

加快职能转变，不断提高政府执行力。按照完善社会主义市场经济体制的要求，把政府主要精力放在为各类市场主体服务上，放在创造良好发展环境上，放在履行社会管理和公共服务职能上。深化行政管理体制改革，推进事业单位改革，推行政企分开、政资分开、政事分开、政府与市场中介组织分开，努力做到权责对等，分工合理，执行顺畅，监督有力。深化行政审批制度改革，进一步加强行政审批中心建设，创新管理机制，完善服务手段，精简审批事项，提高办事效率。提升市长公开电话功能，畅通联系渠道，掌握社情民意，及时解决人民群众反映的问题。

推进依法行政，全面增强政府公信力。贯彻国务院《全面推进依法行政实施纲要》，深化行政执法体制改革，推进相对集中行政处罚权改革，推行行政执法责任制，加强行政复议工作。建立完善行政问责制，加强行政监察与审计监督，认真处理和纠正行政不作为、滥用职权、执法不公、以权谋私等行为。加强政府诚信建设，深化政务公开，完善群众建议征集制度、重大事项社会公示制度、社会听证制度和专家咨询制度，切实保障人民群众对政府工作的知情权、参与权和监督权，提高政府决策的民主化和透明度。自觉接受市人大及其常委会的法律监督和市政协的民主监督，自觉接受群众监督和舆论监督，高度重视人大代表建议和政协提案办理工作。广泛团结各民主党派、工商联、人民团体和社会各界，共同推进物质文明、政治文明和精神文明建设。

保持清正廉洁，进一步树立政府良好形象。树立正确的政绩观，把维护好、实现好、发展好人民群众的根本利益作为一切工作的出发点和落脚点，为群众办实事、解难事、做好事。加强作风建设，大力倡导艰苦奋斗、廉洁从政、真抓实干、服务人民的良好风气。进一步强化教育、制度、监督并重的惩治和预防腐败体系建设，把反腐倡廉工作融入经济建设、政治建设、文化建设、社会建设之中，拓展从源头上防治腐败工作的领域。坚持标本兼治、综合治理、惩防并举、注重预防的方针，抓好领导干部的教育、监督和廉洁自律，抓好大案要案查处，纠正损害群众利益的不正之风。引导各级公务员不断加强自身修养，在实践“八荣八耻”社会主义荣辱观方面做出表率。认真贯彻实施《公务员法》，努力建设一支政治坚定、业务精湛、作风过硬、人民满意的公务员队伍。

各位代表！全面落实科学发展观，加快构建和谐社会，推动经济社会又好又快发展，是各级政府的重要职责和神圣使命。让我们更加紧密地团结在以胡锦涛同志为总书记的党中央周围，高举邓小平理论和“三个代表”重要思想伟大旗帜，在中共烟台市委的坚强领导下，同心同德，开拓进取，为建设文明富庶新烟台而努力奋斗！
